# Bony adaptation signs are predictive of anterior head–neck offset remodeling after internal fixation for slipped capital femoral epiphysis: a multicenter study on 217 patients (228 hips) with follow-up until end of growth

**DOI:** 10.2340/17453674.2025.45076

**Published:** 2026-02-20

**Authors:** Fritz HEFTI, Katharina ODER, Renata POSPISCHILL, Bernd BITTERSOHL, Kathrin LEHNERT, Marco GOETZE, Danimir CERKEZ, Kiril MLADENOV, Bjoern VOGT

**Affiliations:** 1 Children’s University Hospital Basel, Basel, Switzerland; 2 Orthopaedic Hospital Speising, Vienna, Austria; 3 University Hospital Duesseldorf, Duesseldorf; 4 Asklepios Children’s Hospital, St. Augustin; 5 University Hospital Heidelberg, Heidelberg; 6 Olga Children’s Hospital, Stuttgart; 7 Children’s Hospital Altona, Hamburg; 8 University Hospital Muenster, Muenster, Germany

## Abstract

**Background and purpose:**

We aimed to evaluate the prognostic value of radiographic factors in predicting femoral head–neck remodeling, cam deformity, and local growth disturbances in slipped capital femoral epiphysis (SCFE) treated with internal fixation (IF) with or without simultaneous closed reduction.

**Methods:**

A retrospective multicenter study on 217 SCFE patients (228 hips) treated with IF in 10 institutions was performed. Hip morphology was assessed using preoperative, postoperative, and end of growth (EG) radiographs and radial MRI scans. Evaluated parameters included: epi-metaphyseal distance, Southwick angle (SA), head–neck offset (HNO), femoral neck varus deformity, and shortening. Depending on the presence of bony adaptation (BA; defined as rounded anterior metaphyseal edge and posterior callus formation, typically indicating slip onset > 4 weeks) at diagnosis hips were divided into Group A (n = 96; without BA) and Group B (n = 132; with BA).

**Results:**

At EG, Group A demonstrated better slip correction, more favorable femoral neck remodeling, and lower risk of residual cam deformity than Group B (15% vs 58%, risk difference –43%, 95% confidence interval –54 to –32). Remodeling occurred in most cases in both groups, but normal values were not uniformly reached. Femoral neck varus deformity and shortening were observed in both groups and attributed to disease-specific growth plate damage rather than transphyseal fixation.

**Conclusion:**

Radiographic signs of BA at diagnosis can predict the risk of cam deformity at EG. Cases without BA have a favorable prognosis, whereas slips with BA have a higher risk of residual deformity. Signs of BA demonstrated a higher predictive value (0.84) than SA > 40° (0.60) or negative HNO < –5 mm (0.57).

The slipping process in slipped capital femoral epiphysis (SCFE) is a combination of translation and rotation of the femoral head, which usually occurs in the posteromedial direction. The severity of the slip is measured on axial hip radiographs considering only the rotational component using the Southwick angle (SA).

Loder observed radiographic signs of epi-metaphyseal bony adaptation (BA) at diagnosis in 56–84% of cases, comprising “superior and anterior metaphyseal smoothing and inferior and posterior metaphyseal buttressing.” However, the importance of these changes with regard to residual cam deformity was not investigated [1]. Radiographic measurements evaluating the initial translational displacement component of the slip are limited to Klein’s line [2]. However, this was not quantified and the relationship between slip severity and residual cam deformity had not yet been studied. Eijer et al. introduced an “offset” ratio, which was limited to adult patients [3].

Ganz et al. suggested that femoro-acetabular impingement (FAI) may play a central role in the development of early osteoarthritis in nondysplastic hips [4]. The relationship between reduced anterolateral head–neck offset (HNO), FAI, and chondrolabral damage at the acetabular rim was discussed [5,6].

The reported incidence of cam deformity after SCFE at end of growth (EG) is very variable and studies were limited to the preoperative rotational displacement of the femoral head [7-10]. Our study aimed at evaluating the radiographic outcomes and the remodeling between initial presentation and EG in patients with SCFE treated by means of internal fixation (IF). We focused on the assessment of radiographic factors with prognostic importance on remodeling of the head–neck region and the development of cam deformity and growth disturbances.

## Methods

### Study design

We performed a multicenter study on all consecutive cases with SCFE treated by means of IF with or without simultaneous closed reduction of the slip in 10 institutions with prospective data collection. The study is reported according to STROBE guidelines [11].

### Measurements

Bilateral anteroposterior (AP) and frog-lateral hip radiographs were taken in all cases preoperatively, postoperatively, and at EG. EG was defined as bilateral closure of the proximal femoral growth plates. The alpha-angle at EG was measured after implant removal on radial MRI scans [12].

**Figure 1:**
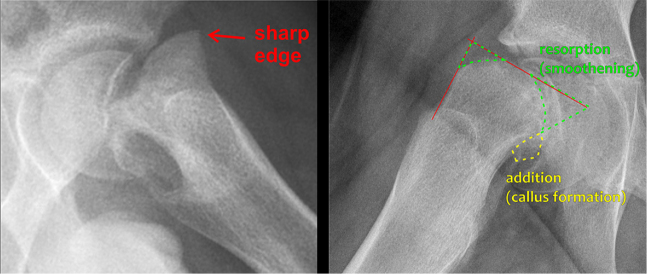
Left: Frog-lateral view demonstrating a severe slip without adaptive bony changes. The sharply delineated anterior metaphyseal edge indicates a recent slip of short duration (Group A). Right: Frog-lateral view demonstrating marked bony adaptations with a rounded anterior metaphyseal contour and callus formation along the posterior femoral neck, consistent with a slip of long duration (Group B).

Hips were classified as “stable” and “unstable” according to Loder [13]. BA was assessed on initial AP and frog-lateral radiographs. Cases without BA demonstrate a characteristic anterior metaphyseal protrusion with well-defined sharp metaphyseal edge, which is a sign of recent onset < 4 weeks (Figure 1 left). BA is defined as rounded anterior metaphyseal edge and posterior callus formation and is typical o slips with onset > 4 weeks (Figure 1 right). Hips with no or only minimal signs of BA and an anterior metaphyseal protrusion > 3 mm were classified as Group A. Hips with signs of BA were allocated to Group B.

Radiographic measurements were performed with the graphic software CorelDRAW V20.0 (Corel Corporation, Ottawa, Canada) [14,15].

**Figure 3:**
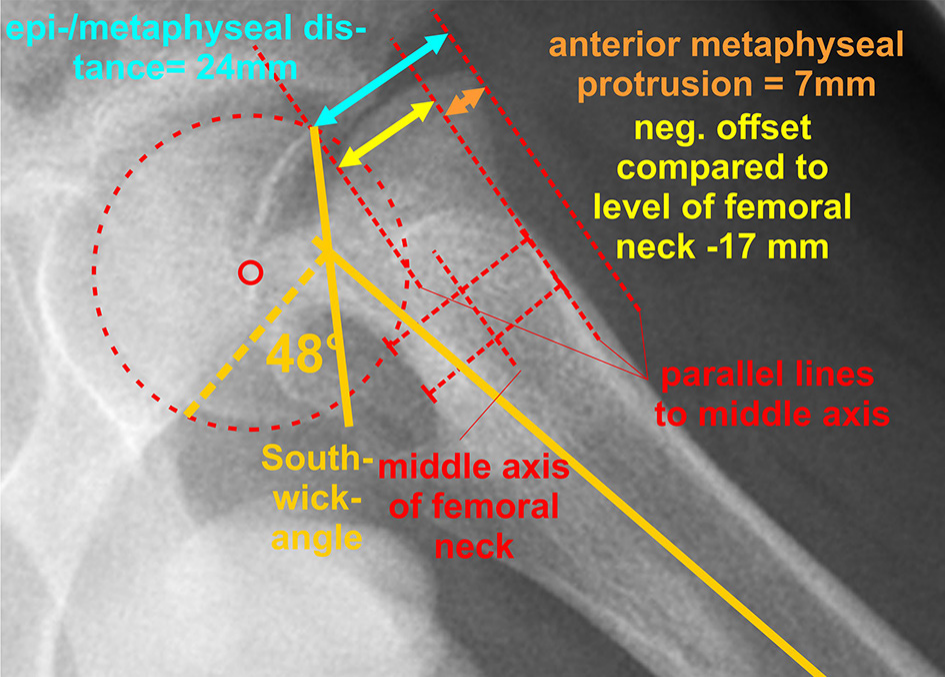
Measurement of the Southwick angle, the epi-metaphyseal distance, the femoral neck offset and the anterior metaphyseal protrusion on a frog-lateral radiograph of a case in Group A. This protrusion can be calculated with the formula epi-metaphyseal distance minus head offset over femoral neck.

SA, HNO, and epi-metaphyseal distance were measured on preoperative, postoperative, and EG frog-lateral radiographs; anterior metaphyseal protrusion (epi-metaphyseal distance minus HNO) on preoperative frog-lateral radiographs (Figure 2), bilateral femoral neck length and width as well as neck-shaft angle on AP, and the alpha-angle according to Nötzli [12] on AP and frog-lateral radiographs at EG (Figures 3–5, see Supplementary data) and on MRI scans. Radiographic measurements were verified in an interrater reliability (IRR) study.

### Operative treatment

IF was performed in all cases, optionally preceded by attempted closed reduction. Implant choice was at the surgeon’s discretion.

### Assessment at end of growth

Final outcome assessment at EG was based on radiographic and clinical parameters, with a “normal hip” serving as the benchmark. The different groups were defined as presented in Table 1. The worst ranked parameter determined the allocation to the respective group.

**Table 1:** Evaluation of the results at end of growth

Classification	Parameters
Normal hip	Alpha-angle < 45°, anterior and lateral head offset > 5 mm of head diameter in all views
Nearly normal hip	Alpha-angle 45°–55° or head offset 3–5 mm in all views
Abnormal hip (cam deformity)	Alpha-angle > 55° or head offset < 3 mm (in any of the radiographs) or pain on flexion/internal rotation and adduction
Disaster	Avascular necrosis or chondrolysis/osteoarthritis because of pin or screw protrusion

### Interrater reliability study

Neck–shaft angle and length–width relation at EG showed very narrow LoA, preoperative SA and alpha-angle moderate LoA and the HNO and epi-metaphyseal distance preoperatively, SA and HNO at EG wider LoA (Table 2).

**Table 2:** LoA in Bland–Altman plots after logarithmic transformation. Values are median LoA with (lower LoA to upper LoAt) and interclass correlation coefficient (ICC)

Factor	Observer 1–Observer 2	Observer 1–Observer 3	Observer 2–Observer 3	ICC	Observer average	Difference LoA max.–min.
SA preoperative	0.00 (–0.14 to 0.14)	0.02 (–0.07 to 0.11)	–0.01 (–0.12 to 0.09)	0.92	0.00 (–0.11 to 0.11)	0.22
Offset preoperative	–0.06 (–0.28 to 0.17)	0.01 (–0.36 to 0.38)	–0.07 (–0.37 to 0.23)	0.68	–0.04 (–0.34 to 0.26)	0.60
Epi-metaphyseal offset	–0.04 (–0.23 to 0.15)	0.00 (–0.28 to 0.28)	–0.04 (–0.23 to 0.16)	0.76	–0.03 (–0.25 to 0.20)	0.44
Neck–shaft angle	0.00 (–0.03 to 0.04)	0.00 (–0.02 to 0.03)	0.00 (–0.04 to 0.04)	0.79	0.00 (–0.03 to 0.04)	0.07
Length–width relation	0.01 (–0.05 to 0.07)	0.02 (–0.04 to 0.08)	–0.01 (–0.08 to 0.06)	0.76	0.01 (–0.06 to 0.07)	0.13
Alpha–angle	–0.02 (–0.16 to 0.11)	–0.05 (–0.18 to 0.07)	0.03 (–0.08 to 0.13)	0.72	–0.01 (–0.14 to 0.10)	0.24
SA at EG	0.06 (–0.13 to 0.25)	0.10 (–0.19 to 0.39)	–0.04 (–0.39 to 0.31)	0.82	0.04 (–0.24 to 0.32)	0.55
Offset at EG	–0.03 (–0.35 to 0.29)	0.06 (–0.12 to 0.24)	–0.09 (–0.41 to 0.24)	0.84	–0.02 (–0.29 to 0.26)	0.55

LoA: limits of agreement, SA: Southwick angle.

### Statistics

The positive predictive values at the preoperative frog-lateral view for cam deformity for BA, SA > 40° and HNO < –5 mm were determined. Sensitivity and specificity were calculated for various preoperative parameters chosen after a receiver operating characteristic curve analysis.

For the relevant measurements, an IRR study was conducted. 3 observers (FH, BV, and KM) independently measured 8 parameters each in 20 identical cases from the cohort. Bland–Altman analyses were performed between each pair of reviewers. The limits of agreement (LoA) after logarithmic transformation were determined, and the mean of the 3 pairwise comparisons was calculated.

“R4.3.3.” (R Foundation for Statistical Computing Vienna, Austria) and “DATAtab” (https://numiqo.com/) were used for statistical calculations. Categorical variables were presented in absolute and relative numbers. For continuous variables normal distribution was checked with quantile–quantile plots and a Kolmogorov–Smirnov test. For normally distributed metric parameters, mean values were stated with standard deviation (SD). Mean values were compared using a t test for independent samples. For non-normally distributed metric data, median values were stated with interquartile range (IQR) presented as 25th–75th percentile. Median values were compared using a Mann–Whitney U test for unpaired samples or Wilcoxon signed-rank test for paired samples. A 95% confidence interval (CI) was chosen, and the significance level was set at α < 0.05. For analysis of contingency tables Pearson’s chi-square test was used. A multiple regression analysis was conducted to evaluate the differences between the postoperative values and those at EG. All statistical analyses were revised by a professional medical statistician. The analyses were done naively assuming that the hips of uni- and bilaterally affected patients could be handled as if they were independently distributed.

### Ethics, data sharing plan, funding, use of AI, and disclosures

The study has been performed in accordance with the ethical standards in the 1964 Declaration of Helsinki and the US Health Insurance Portability and Accountability Act (HIPAA). Each hospital had the approval of the local ethical committee for the conduct of this study.

The study was funded by the “Deutsche Arthrosehilfe” and the “Vereinigung Kinderorthopaedie”.

AI tools were not used.

Complete disclosure of interest forms according to ICMJE are available on the article page, doi: 10.2340/17453674.2025.45076

## Results

### Basic data

**Figure 6:**
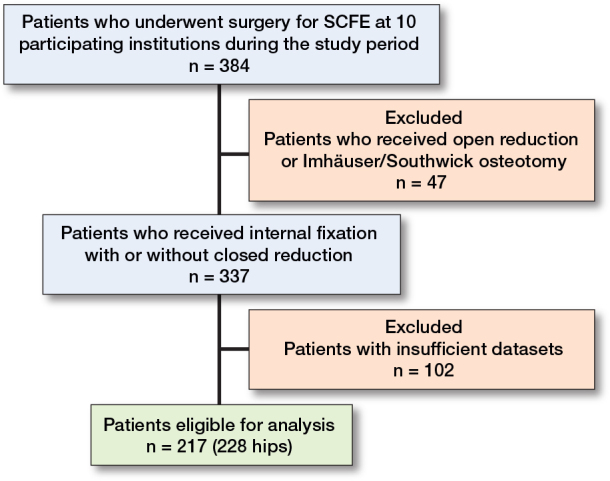
Patient flow diagram showing the inclusion and exclusion criteria in this study.

The study group comprised 217 patients with 228 affected hips (Figure 6). Patient demographics are listed in Table 3.

**Table 3:** Patient demographics. Values are presented as numbers, numbers (percentage), or median (interquartile range).

Factor	All patients	Group A	Group B
Number of patients **^a^**	217	95	122
Bilateral slip	11 (5)	1 (1)	10 (8)
Unilateral slip	206 (95)	94 (99)	112 (92)
Number of affected hips **^a^**	228	96	132
Sex (patients)			
Male	101 (47)	39 (41)	62 (51)
Female	116 (53)	56 (59)	60 (49)
Age at presentation (years)	12.9 (7.2–17.2)	12.3 (8.2–15.3)	13.0 (7.2–17.2)
Male	12.9 (8.6–17.1)	12.3 (10.0–14.6)	13.3 (8.1–17.1)
Female	12.5 (7.2–17.2)	12.3 (8.2–15.3)	12.7 (7.2–17.2)
Walking ability (patients)			
Able to walk (stable)	185 (85)	80 (84)	105 (86)
Unable to walk (unstable)	32 (15)	15 (16)	17 (14)
Weight (patients)			
Normal (BMI < 25)	146 (67)	65 (68)	81 (67)
Overweight (BMI 25–30)	43 (20)	18 (19)	25 (20)
Severe obesity (BMI > 30)	28 (13)	12 (13)	16 (13)
Affected side (hips)			
Right	86 (38)	31 (32)	55 (42)
Left	142 (62)	65 (68)	77 (58)
Stability (hips)			
Stable	186 (82)	76 (79)	110 (83)
Unstable	42 (18)	20 (21)	22 (17)
Type of internal fixation (hips)			
Pins	74 (32)	35 (37)	39 (30)
Hansson pins	22 (10)	7 (7)	15 (11)
Screws	132 (58)	54 (56)	78 (59)
Short thread	96 (73)	39 (72)	57 (73)
Fully threaded	36 (27)	15 (28)	21 (27)
Fixation at the unaffected side **^b^**	198 (96)	89 (95)	109 (97)
Follow-up time (months)	46 (24–164)	48 (24–164)	45 (24–156)
Hips with removed implants at EG	144 (63)	54 (56)	90 (68)
Hips with MRI scan at EG	142 (62)	61 (64)	81 (61)

BMI: body mass index, EG: end of growth.

a with IF and complete data set.

b in patients with unilateral slip, always with identical method as on the affected side.

96 of the 228 hips (42%) in 94 patients demonstrated no or minimal signs of BA and had an anterior metaphyseal protrusion of > 3 mm of the femoral head diameter at initial presentation (Group A). 132 hips (58%) in 112 patients showed BA (Group B). Patients in Group A were significantly younger at diagnosis with a mean age of 12.3 years (IQR 8.2–15.3) than those in Group B at a mean of 13.0 years (IQR 7.2–17.2).

### Evolution of the Southwick angle and the anterior femoral head–neck offset

The initial correction of HNO after closed reduction in Group A was better than in Group B (3.0 mm [CI 1.3–4.7] and 1.5 mm [CI 0.8–3.8]), in which more remodeling occurred with follow-up (3.1 mm [CI 1.9–4.4] in Group A and 8.0 mm [CI 5.9–8.2] in Group B) (Table 4). Remodeling was observed in most of the cases in both groups; however, normal ranges were not uniformly reached [16]. The difference between the median values preoperatively, postoperatively, and at EG was statistically significant in both groups (P < 0.001).

**Table 4:** Measurements on frog-lateral radiographs preoperatively, postoperatively, and at EG in Groups A and B

Factor	Group A (n = 96)	P value	Group B (n = 132)	P value
Southwick angle, median (IQR) degrees				
Preoperative	30.0 (24.0 to 39.0)	–	42.0 (33.8 to 54.0)	–
Postoperative	21.0 (16.0 to 27.0)	–	35.0 (26.0 to 43.0)	–
At EG	16.5 (12.3 to 23.0)	–	26.0 (19.0 to 29.0)	–
Differences, median (CI)				
Preoperative–postop.	9.0 (6.0 to 13.0)	< 0.001	7.0 (4.5 to 13.0)	< 0.001
Postoperative–EG	4.5 (0.5 to 7.0)	< 0.01	9.0 (5.0 to 12.0)	< 0.001
Preoperative–EG	13.5 (10.0 to 17.0)	< 0.001	16.0 (14.0 to 20.0)	< 0.001
Head–neck offset, median (IQR) mm				
Preoperative	0.0 (–2.7 to 3.4)	–	–6.3 (–13.4 to –2.2)	–
Postoperative	3.0 (0.6 to 7.1)	–	–4.8 (–9.5 to –0.6)	–
At EG	6.1 (5.3 to 7.0)	–	3.2 (–2.5 to 7.3)	–
Differences, median (CI)				
Postoperative–preop.	3.0 (1.3 to 4.7)	< 0.01	1.5 (−0.8 to 3.8)	< 0.001
EG–postoperative	3.1 (1.9 to 4.4)	< 0.001	8.0 (5.9 to 8.2)	< 0.001
EG–preoperative	6.1 (4.5 to 7.0)	< 0.001	9.5 (9.3 to 13.2)	< 0.001

IQR: interquartile range. CI: 95% confidence interval. EG: end of growth.

Multiple regression analysis of the values for SA and HNO postoperatively and at EG showed a statistically highly significant difference between Group A and Group B (P < 0.001) and a moderately significant dependence of the correction on age (P < 0.05), while sex showed no influence (P > 0.05).

### Outcomes for residual cam deformity related to preoperative findings

**Figure 7:**
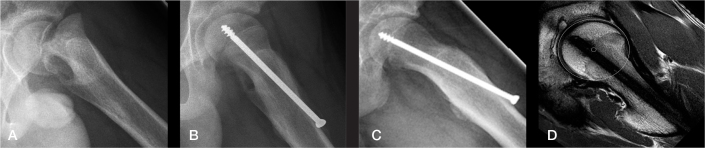
Illustrative case from Group A (frog-lateral radiographs). A) Preoperative radiograph. B) Postoperative radiograph. C) Radiograph at end of growth (EG). D) MRI at EG showing normal anterior head–neck offset (same patient as in Figure 1 left).

**Figure 8:**
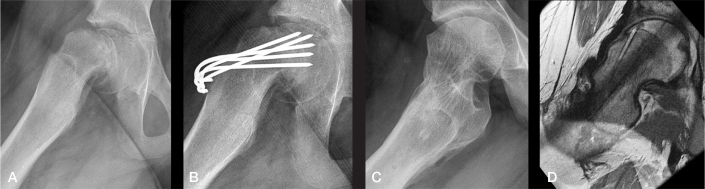
Illustrative case from Group B (frog-lateral radiographs). A) Preoperative. B) Postoperative after internal fixation. C) Radiograph at end of growth (EG). D) MRI at EG. A marked cam deformity is present at EG with an alpha-angle of 80° (same patient as in Figure 1 right).

Regarding cam deformity, statistically significant differences between Group A and B (P < 0.001) were observed with a significantly higher percentage in Group B than in Group A (15% vs 58%, risk difference –43%, CI –54 to –32). Both, a more negative initial HNO (> –5 mm) and a larger SA (> 40°) showed a negative effect on the prognosis concerning cam deformity (Table 5). All hips presenting with a cam deformity at EG exhibited an alpha angle > 55°, as detected on MRI (available in 142 cases [62%]) and/or in at least 1 of the radiographic projections. Illustrative cases from Groups A and B are presented in Figures 7 and 8.

**Table 5:** Result at end of growth depending on radiographic measurements at initial presentation. Values are count (percentage)

Factor	Preoperative findings	Result at end of growth ^a^			
		Normal	Nearly normal	Abnormal	Disaster
All cases	228	84 (37)	48 (21)	89 (39)	7 (3.1)
Group A	96 (42)	56 (58)	24 (25)	14 (15)	2 (2.1)
SA < 40°	61	37	16	7	1
HNO > –5 mm	60	37	16	6	1
HNO ≤ –5 mm	1	0	0	1	0
SA ≥ 40°	35	19	8	7	1
HNO > –5 mm	29	19	7	2	1
HNO ≤ –5 mm	6	0	1	5	0
Group B	132 (58)	28 (21)	24 (18)	75 (57)	5 (3.8)
SA < 40°	56	13	13	28	2
HNO > –5 mm	41	8	12	19	2
HNO ≤ –5 mm	15	5	1	9	0
SA ≥ 40°	76	15	11	47	3
HNO > –5 mm	23	8	2	11	2
HNO ≤ –5 mm	53	7	9	36	1

a See Table 1 for description of classification.

SA: Southwick angle, HNO: head–neck offset.

### Predictive values for a cam deformity depending on the initial status

The predictive values of 3 criteria regarding the risk for cam deformity at EG were investigated. The presence of BA showed a very high predictive value of 0.88 (CI 0.75–0.91). The predictive value of SA of > 40° (0.60, CI 0.49–0.70]) or HNO of < –5 mm (0.57. CI 0.46–0.67) was moderate (Table 6).

**Table 6:** Predictive values for residual cam deformity at EG with (95% confidence interval)

Factor	Positive predictive value	Negative predictive value	Sensitivity	Specificity
Presence of BA	0.84 (0.77–0.92)	0.61 (0.52–0.69)	0.59 (0.51–0.68)	0.85 (0.78–0.92)
SA > 40°	0.60 (0.49–0.70)	0.33 (0.23–0.43)	0.50 (0.41–0.60)	0.48 (0.35–0.62)
HNO < –5 mm	0.57 (0.46–0.67)	0.83 (0.76–0.98)	0.70 (0.58–0.80)	0.74 (0.67–0.81)

SA: Southwick angle, BA: bony adaptation, HNO: head–neck offset.

### Neck–shaft angle and shortening of the femoral neck

At EG a decreased neck–shaft angle (varus deformity) was observed on the affected side as compared with the unaffected side. This was more significantly pronounced in Group B (Cliff’s Delta 0.82, CI 0.75–0.88) than in Group A (Cliff’s Delta 0.26, CI 0.12–0.40). There was no difference between the various implants used (P > 0.05). The neck was significantly shorter on the affected than the unaffected side; this was more pronounced in Group B (Cliff’s Delta 0.66, CI 0.55–0.75) than in Group A (Cliff’s Delta 0.45, CI 0.32–0.57) and was independent of the implant used (P > 0.05). Neck shortening was not influenced by patient age at initial diagnosis. The difference in shortening in the age group below and above the median age of 12.9 years was not statistically significant (P = 0.6). Data for neck varus deformity and shortening is summarized in Tables 7 and 8.

**Table 7:** Measurements on AP radiographs at EG in Group A and B and the unaffected side with prophylactic internal fixation

Group	Neck–shaft angle		Relation width/length of femoral neck	
Type of internal fixation	n	mean (IQR)	n	mean (IQR)
A All	96	131 (126–135)	96	1.3 (1.2–1.4)
Pins	35	132 (128–139)	35	1.3 (1.2–1.4)
Hansson pins	7	127 (121–132)	7	1.3 (1.2–1.5)
Short threaded screws	39	131 (124–135)	39	1.3 (1.2–1.4)
Fully threaded screws	15	127 (121–132)	15	1.3 (1.2–1.3)
B All	132	121 (111–127)	132	1.2 (1.1–1.3)
Pins	39	122 (114–128)	39	1.2 (1.0–1.3)
Hansson pins	15	123 (118–127)	15	1.3 (1.2–1.4)
Short threaded screws	57	119 (110–125)	57	1.2 (1.1–1.3)
Fully threaded screws	21	120 (112–128)	21	1.1 (1.0–1.2)
Unaffected side				
All	197	134 (130–138)	193	1.5 (1.3–1.6)
Pins	71	135 (130–137)	69	1.5 (1.4–1.6)
Hansson pins	16	130 (127–133)	16	1.5 (1.4–1.6)
Short threaded screws	85	135 (131–140)	84	1.5 (1.3–1.5)
Fully threaded screws	25	135 (131–135)	24	1.5 (1.3–1.5)

IQR: interquartile range

**Table 8:** Evaluation of the differences between the groups (all). Values are median (95% confidence interval)

Differences	Neck–shaft angle		Relation width/length of femoral neck	
	Cliff’s Delta	P value	Cliff’s Delta	P value
Group A–Group B	0.62 (0.50–0.72)	< 0.001	0.32 (0.18–0.46)	< 0.001
Unaffected side–Group A	0.26 (0.12–0.40)	< 0.001	0.45 (0.32–0.57)	< 0.001
Unaffected side–Group B	0.82 (0.75–0.88)	< 0.001	0.66 (0.55–0.75)	< 0.001

The differences between the subgroups of the different types of internal fixation were not significant (P >0.05).

### Complications and revision surgeries

Complications are presented in Table 9. In 3 of the 5 cases with avascular necrosis (AVN) of the femoral head the slip was unstable.

**Table 9:** Complications and revision surgeries

Complications	Group A	Group B	Un-affected side	Total
Avascular necrosis	1	4		5
Intraarticular perforation of implants in affected hip	1	2	1	4
Osteoarthritis after implant perforation	1	1		2
Subtrochanteric fracture (after use of Hansson pins)	1	1	1	3
Delayed wound healing	2	1		3
Pin fracture	1	1		2
Revision surgeries before the end of growth				
Exchange of pins	1	1	2	4
Exchange of screws	6	5	11	22
Switching from pins to screws	7	4	11	22
Internal fixation because of fracture	1	1	1	3
Surgery after the end of growth				
Arthroscopic trimming for cam deformity	1	9		10
Secondary Imhäuser–Southwick osteotomy	5		5	
Total hip replacement		2		2

Surgeries for elective implant removal at end of growth were excluded.

## Discussion

We aimed to evaluate the prognostic value of radiographic factors in predicting femoral head–neck remodeling, cam deformity, and local growth disturbances in SCFE treated with IF with or without simultaneous closed reduction. We found that radiographic signs of BA at diagnosis can predict the risk of cam deformity at EG. Cases without BA have a favorable prognosis, whereas slips with BA have a higher risk of residual deformity. Signs of BA demonstrated a higher predictive value (0.84) than SA > 40° (0.60) or negative HNO < –5 mm (0.57).

Limited knowledge exists concerning the remodeling potential in SCFE patients treated by means of IF. Akiyama et al. reported on 69 hips of which 29.4% developed cam deformity [7]. Klit et al. found cam deformity in 10 of 24 hips (41.6%) [9]. Nectoux et al. identified cam deformities in only 15 of 222 patients (6.7%) [17]. Recently Andersen et al. reported good initial correction after IF; however, further correction of the SA was limited [18]. Our results are concordant with the observations regarding initial correction. However, we observed greater additional correction of the anterior femoral HNO with further follow-up. As our study aimed to assess the influence of residual growth on femoral neck remodeling, follow-up was extended only until EG.

To our knowledge, the severity of initial translation of the femoral head in relation to the femoral neck at the time of slip diagnosis has not yet been considered in the prognostic assessment of SCFE patients. In particular, the measurement of the anterior metaphyseal protrusion is a useful tool to assess presence or absence of BA. The recognition of BA contributes importantly to the assessment of the duration of the slipping process preceding the diagnosis of SCFE and has a reliable prognostic value concerning remodeling potential and the risk of residual cam deformity. Patients with anterior neck metaphyseal protrusion of ≥ 3 mm and radiologically sharply defined bony contour have a good prognosis and low risk of residual cam deformity.

Akiyama et al. used a similar measurement (after EG) and found an even higher incidence of pathological hips compared with the measurements of the alpha-angle on MRI scans [7]. Our IRR study showed a moderate reliability of these measurements. In our study MRI scans were uniformly performed after implant removal to avoid misinterpretation of the alpha-angle due to oval distortion of the head caused by implant-related artifacts. The limits of the alpha-angle regarding the presence of cam deformity are controversially discussed. Pollard et al. demonstrated that most patients with cam impingement have an alpha-angle >63° [19], whereas Nötzli et al. considered 50° as the limit [12]. In consideration of these restrictions, we postulate that our results are valid, which is supported by the large number of patients.

Median SA and HNO improved significantly directly after surgery. These findings indicate that reduction of the slip occurred to some extent in the majority of cases. Slip reduction was better in Group A than in Group B. However, it remains unclear how many of the reductions were achieved intentionally through a specific maneuver and how many occurred spontaneously because of patient positioning. BA of the head–neck region has been described by Loder [1]. We focused our assessment on the predictive value of metaphyseal changes and found significant correlations to the development of cam deformity. Patients without BA at initial presentation (Group A) have a very good prognosis concerning residual cam deformity. Contrary to this, patients showing BA on initial presentation (Group B) had > 50% risk of residual cam deformity at EG. In a consensus paper, IF was considered to be the method of choice for SCFE patients with all types of mild slips (stable and unstable) and in those with moderate stable slips [20]. This treatment approach is consistent with the results of a prospective cohort study based on the total Swedish national population, which showed that open reduction or femoral neck recontouring is very rarely performed and not routinely recommended, but is reserved for exceptional cases with unstable or severe slips [21]. Our study shows that the assessment of signs of BA has an even more important value than the SA regarding the risk of residual cam deformity, which is relevant for the choice of the surgical procedure.

The best imaging method to identify cam deformity is a radial MRI scan as described by Nötzli et al. [12]. As the cam bump is located anterolaterally it is not always visible on AP or frog-lateral hip radiographs. Cam deformity was observed in 40% of the affected hips that received MRI scan at EG (56 of 142 hips). A similar percentage (39%) was observed in the group that received only plain radiography at EG. Only in 2 of 76 abnormal hips (2.6%) were MRI scans the only imaging procedure for detecting cam deformity. We can presume that most cam deformities could be detected on native frog-lateral radiographs and clinical assessment of internal hip rotation [22,23].

An interesting observation was the significantly decreased neck–shaft angle in the affected hips compared with the unaffected side and the decreased neck length-to-width ratio, which was interpreted as an indirect sign of neck shortening. This phenomenon was particularly observed in Group B hips with a longer duration of the disease before diagnosis. As the unaffected hip was prophylactically addressed with IF in 96% of the unilateral cases with the same implant type as the affected hip, this effect is clearly a result of disease-related damage to the growth plate and not a consequence of the transphyseal fixation. Regarding the effect on femoral neck growth, we did not find significant differences between the various types of implants used for IF in our cohort, which supports the statement that the shape of the implant has no effect on the development of neck deformity. Comparison with non-operated normal control hips was not possible due to the insufficient number of hips without IF. Deceleration of physeal growth by means of screw epiphysiodesis occurs through compression across the growth plate with bicortical screw insertion or oblique positioning in relation to the epiphyseal plate. When using screw fixation in SCFE, no relevant physeal compression effect occurs because the screw’s thread lies in cancellous bone, and its axis is perpendicular to the growth plate. In our study no implant-related proximal femoral growth disturbance was observed provided that the growth plate was initially not affected, and the screw was inserted perpendicular to the growth plate.

In our series, AVN was observed in 2.2%, rising to 7.1% among initially unstable hips. This rate is lower than in other reports on AVN in unstable hips [24] and can be hypothetically explained by the fact that a certain number of the hips at risk of AVN underwent an open reduction at initial treatment and were not included in this study.

### Limitations

The treatment method (IF with or without closed reduction vs open reduction) was according to the surgeon’s preference, which is a potential source of selection bias. Potential inaccuracies of the measurements should be taken into consideration. In a femur model the accuracy of the measurement of the SA on frog-lateral radiographs was within a range of ±10°, if the leg was not more than 30° externally rotated [25]. This was essentially confirmed in a recent computed tomography study [26]. In the preoperative radiographs the edges of the epiphysis of the femoral head are usually sharp and allow precise determination of the axis of the proximal femoral epiphysis (and the SA). After EG, however, it is much more difficult to clearly define the margins of the epiphysis. In offset measurements, the difficulty lies in determining the neck axis, especially when BA are present and the neck is bent. This may affect measurement accuracy. The standardized measurement techniques at EG applied in the present work are described in the Supplementary data (see Figures 4–6).

### Conclusion

We found that radiographic signs of BA at diagnosis can predict the risk of cam deformity at EG. Cases without BA have a favorable prognosis, whereas slips with BA have a higher risk of residual deformity. Signs of BA demonstrated a higher predictive value (0.84) than SA > 40° (0.60) or negative HNO < –5 mm (0.57).

*In perspective*, SCFE patients without signs of BA at the time of initial diagnosis have a favorable prognosis regarding development of cam deformity. However, all patients with SCFE develop femoral neck varus deformity and shortening, primarily due to disease-related damage to the growth plate, but also as a result of the slip itself and subsequent remodeling. This occurs irrespective of the implant type used. These effects are significantly more pronounced in cases with signs of BA at initial presentation. Physeal growth disturbances are not observed on the identically treated unaffected side.

### Supplementary data

Figures 4–6 are availabe as Supplementary data on the article homepage, doi: 10.2340/17453674.2025.45076

## Supplementary Material



## Notes

FH: conceptualization, data curation, formal analysis, funding acquisition, investigation, methodology, project administration, resources, supervision, validation, visualization, writing—original draft, writing—review and editing. BB, DC, KL, KO, MG, RP: data curation, investigation, writing—review and editing. BV, KM: conceptualization, data curation, formal analysis, methodology, writing—original draft, writing—review and editing

The authors would like to thank the following people for supporting this study as heads of the participating clinical units: Prof. Dr. Carol Hasler, University Children’s Hospital Basel (UKBB), Basel, Switzerland; Prof. Dr. Thomas Wirth, Olgahospital, Stuttgart, Germany; Prof. Dr. Ralf Stuecker, Children’s Hospital Altona, Hamburg, Germany; Prof. Dr. Robert Roedl, University Hospital Muenster, Muenster, Germany; Priv.-Doz. Dr. Urs von Deimling, Asklepios Children’s Hospital, St. Augustin, Germany; Prof. Dr. Ruediger Krauspe, University Hospital Duesseldorf, Duesseldorf, Germany; Prim.-Doz. Dr. Rudolf Ganger, PhD, Orthopaedic Hospital Speising, Vienna, Austria; Primaria Dr. Walpurga Lick-Schiffer, Orthopaedic Hospital Stolzalpe, Stolzalpe, Austria. They would also like to thank these colleagues who have also contributed and entered cases: Dr. Kerstin Remmel, Cnopf Children’s Hospital, Nuremberg, Germany; Dr. Christian Tschauner, Ass. Prof., Orthopaedic Hospital Stolzalpe, Stolzalpe, Austria. They also thank the following people for their help: Dr. Zdzislav Krol, PhD, University Children’s Hospital Basel (UKBB), Basel, Switzerland, who was responsible for the website with the database; Dr. Helmut Huberti and Mr. Ingo Tonner from the “Deutsche Arthrosehilfe” for financial support; Prof. Dr. Lutz Jani †, who established the connection between the “Deutsche Arthrosehilfe” and the “Vereinigung Kinderorthopaedie”; Mrs Mandy Webb for the linguistic polish; Ms Sabine Schaedelin, MSc for the support in the statistical analysis.

Handling co-editors: Ilkka Helenius

*Acta* thanks Gunnar Hägglund, Randall Loder and Terje Terjesen for help with peer review of this manuscript.
