# Supplementary material for: Bony adaptation signs are predictive of anterior head–neck offset remodeling after internal fixation for slipped capital femoral epiphysis: a multicenter study on 217 patients (228 hips) with follow-up until end of growth
Source: Acta Orthop. 2026 Feb 20;97:117–25. doi: 10.2340/17453674.2025.45076 (PMC12922479; doi:10.2340/17453674.2025.45076)
Supplement: Supplementary file 1 [file ActaO-97-45076-s1.pdf]

Supplementary Material

Figure 3

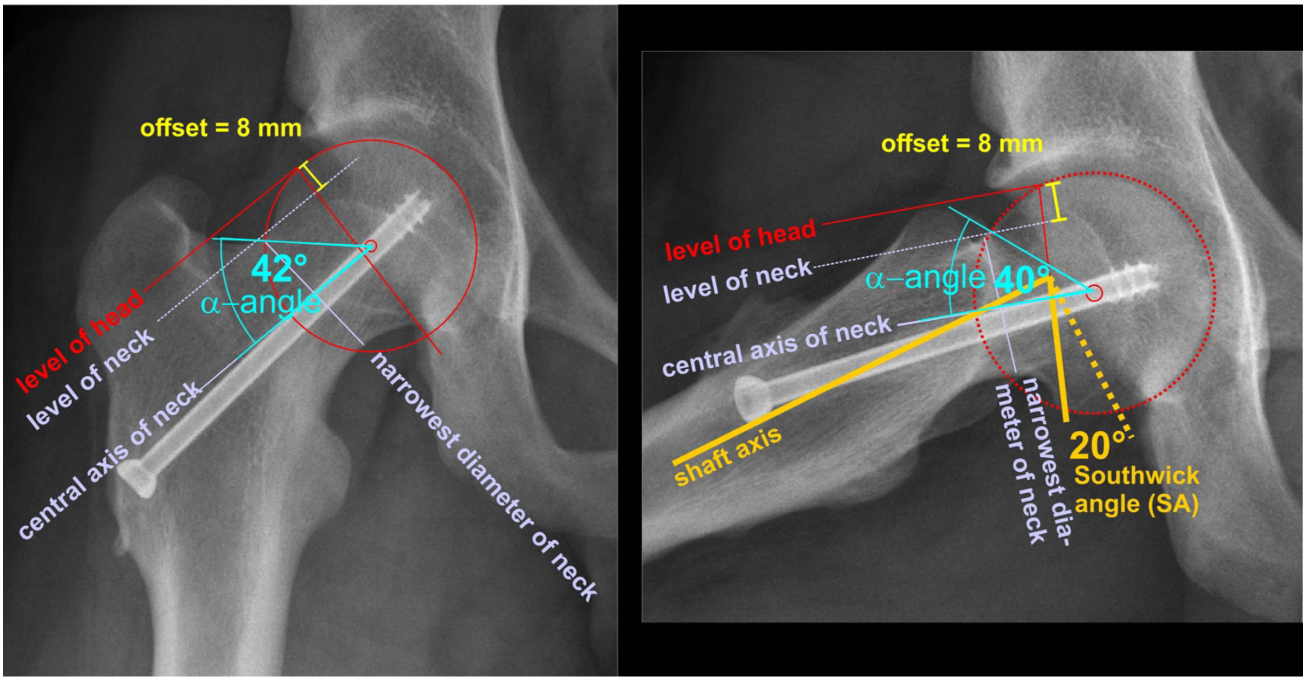

Measurements of the Southwick angle (SA), the positive offset and the alpha-angle at end of growth in cases of a normal position of the head on anteroposterior **(left)** and frog-lateral radiographs **(right)** in a case with a centered head.

Figure 4

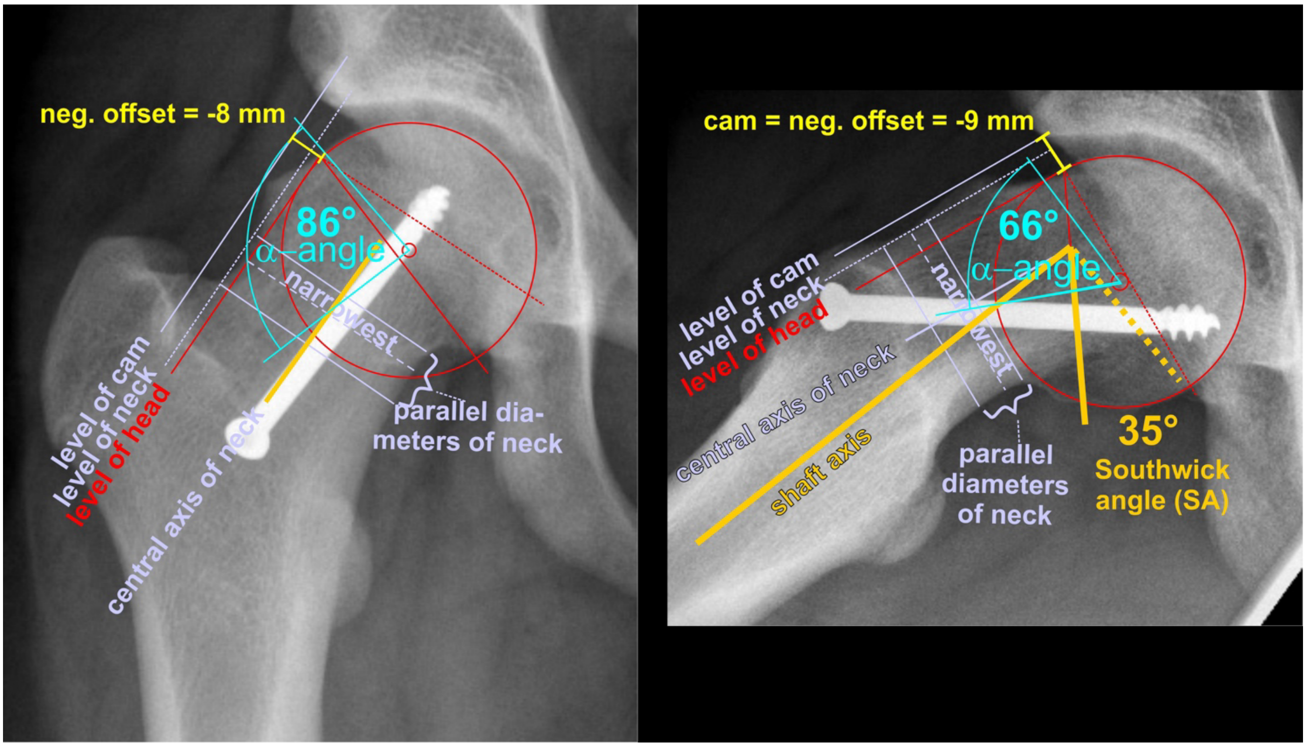

Measurements of the Southwick angle (SA), the positive offset and the alpha-angle at end of growth in cases of a decentered head on anteroposterior **(left)** and frog-lateral radiographs **(right)** in a case with a centered head.

Figure 5

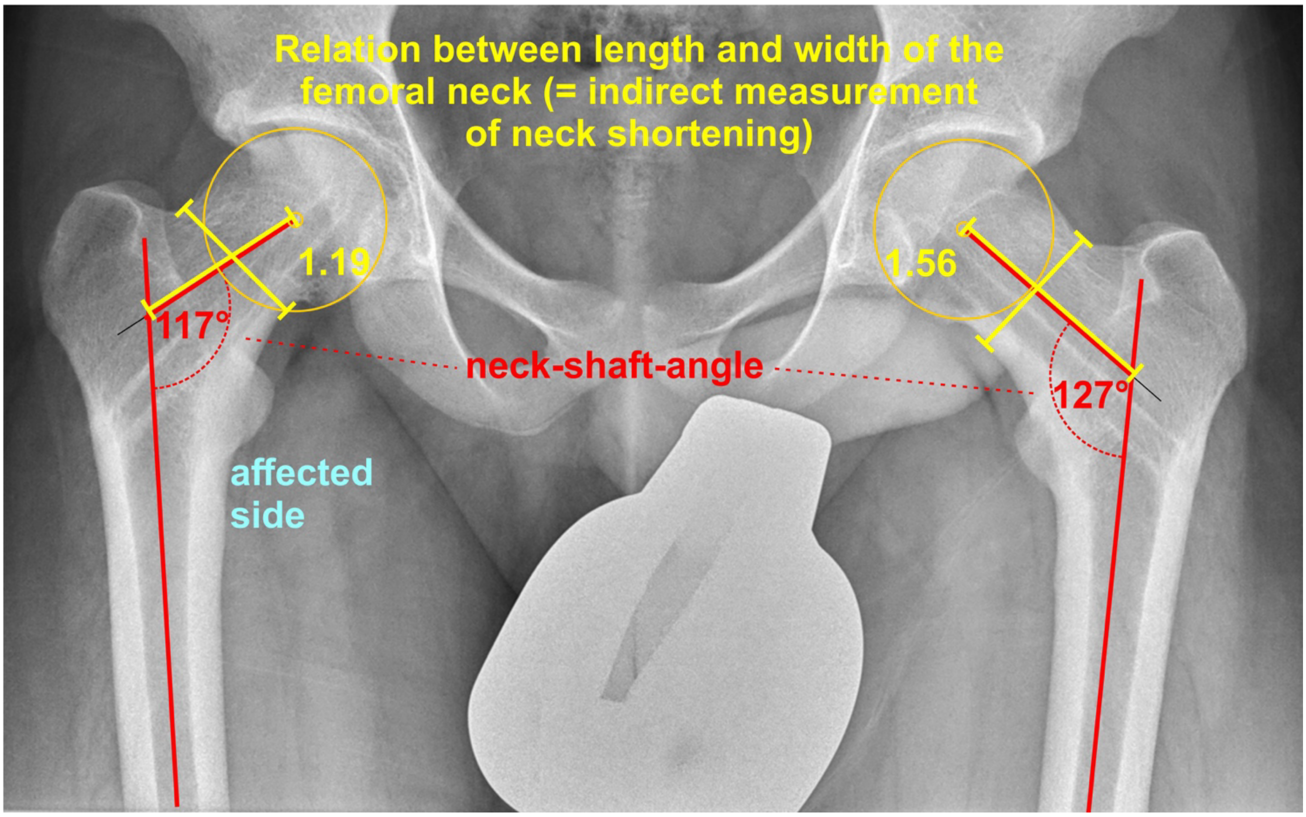

At end of growth the neck-shaft angle and the relation between length and width of the femoral neck was measured with a standardized method. This radiograph is also an example of the growth disturbance on the affected side.
